# Ribosomal protein L18aB is required for both male gametophyte function and embryo development in *Arabidopsis*

**DOI:** 10.1038/srep31195

**Published:** 2016-08-09

**Authors:** Hailong Yan, Dan Chen, Yifan Wang, Yang Sun, Jing Zhao, Mengxiang Sun, Xiongbo Peng

**Affiliations:** 1 State Key Laboratory for Hybrid Rice, College of Life Sciences, Wuhan University, Wuhan 430072, China

## Abstract

Ribosomal proteins are involved in numerous essential cell activities in plants. However, the regulatory role in specific plant developmental processes has not yet been fully elucidated. Here we identified the new ribosomal protein L18aB, which is specifically involved in sexual reproduction and plays a critical role in male gametophyte development and embryo pattern formation. In *rpl18aB* mutant plants, the mature pollen grains can germinate normally, but their competitiveness for growing in the style is significantly reduced. More interestingly, RPL18aB is required in early embryogenesis. *rpl18aB* embryos displayed irregular cell division orientations in the early pro-embryo and arrested at the globular stage with possible, secondary pattern formation defects. Further investigations revealed that the polar transportation of auxin is disturbed in the *rpl18aB* mutant embryos, which may explain the observed failure in embryo pattern formation. The cell type-specific complementation of *RPL18aB* in *rpl18aB* was not able to recover the phenotype, indicating that RPL18aB may play an essential role in early cell fate determination. This work unravels a novel role in embryo development for a ribosomal protein, and provides insight into regulatory mechanism of early embryogenesis.

In plants, ribosomal proteins are encoded by small gene families. The *Arabidopsis thaliana* genome encodes for approximately 80 cytoplasmic ribosomal proteins, and each protein is encoded by 2–7 family members since different genes within a family share 65–100% amino acid sequence identity^1^. The presence of multiple genes for each ribosomal protein in plants might be necessary to maintain constant ribosomal protein amounts or some degree of ribosome heterogeneity and functional specialization. Phenotypes resulting from mutations in several different ribosomal protein genes provide strong evidence that ribosomes participate as regulatory components of developmental pathways^2^.

Although ribosomes are ubiquitously distributed in the plant and are essential for plant development, many ribosomal protein loss-of-function mutants are viable. These mutants typically display a subtle change in leaf shape^3^,^4^,^5^,^6^,^7^,^8^,^9^. These observations appear to indicate that many ribosomal proteins must be functionally specialized for a precise developmental process, thus their loss-of-function does not affect all cellular processes. Furthermore, it was recently demonstrated that ribosomal proteins control auxin mediated developmental programs by translational regulation of auxin response factors^10^. Together these findings indicate that the specific role of each ribosomal protein in plant development must be studied individually, despite their general function being well known.

Previous studies also indicated that ribosomes play an important role in sexual plant reproduction. Loss-of-function mutations in single ribosomal protein genes could result in female gametophyte defects. Mutations in the ribosomal protein genes RPL24B, RPL10A, RPS5A and in the ribosomal protein RPL27aC reduce female fertility^11^,^12^,^13^,^14^. Mutations in seven cytoplasmic ribosomal proteins, S6, S11, L2, L8, L23, L19 L40 and RPL27a, resulted in embryo developmental defects^14^,^15^. In these mutants, the earliest divisions of the zygote forming a suspensor and a globular stage embryo appear to be unaltered, indicating that early patterning events are unaffected by the loss of these ribosomal proteins. These ribosomal proteins are required for the development of the embryo beyond the globular stage. Early-embryo development specific ribosomal proteins and ribosomal proteins involved in other processes of sexual plant reproduction remain to be identified.

Here, we isolated the new ribosomal protein gene *RPL18aB.* The Loss of function of this gene result in weakened function of male gametophyte and abortion of early embryo development, with a striking alteration in early embryo patterning, indicating the essential role of RPL18aB in these developmental processes.

## Results

### A T-DNA insertion in RPL18aB affects seed development

**Figure 1:**
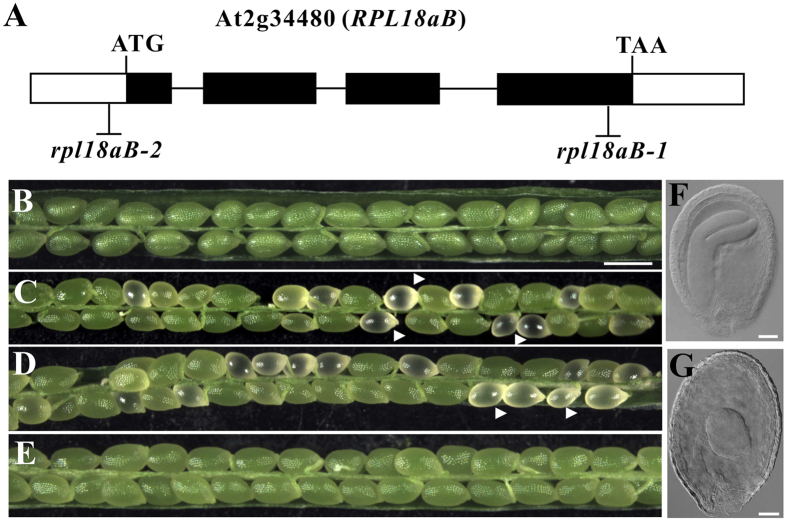
Embryo developmental processes are impaired in *rpl18aB* plant. (**A**) Diagram of the *RPL18aB* genomic DNA and the insertion sites for the T-DNA. The *RPL18aB* gene (AT2g34480) has four exons. The T-DNA inserted into the fourth exon in *rpl18aB-1* and the T-DNA was inserted into the promoter at 23 bp upstream of the ATG start codon in *rpl18aB-2*. Micrographs of siliques of wild type (**B**), *rpl18aB-1* plant (**C**), *rpl18aB-2* plant (**D**) and genomic complemented line (**E**). Silique of wild type and genomic complemented line show only green seeds (**B,E**), while *rpl18aB* plant silique showing normal green seeds and white seeds indicated by arrow head (**C**,**D**). (**F**,**G**) Micrographs of seeds of *rpl18aB-1* plant. In the *rpl18aB-1* plant, when wild type embryo develops totorpedo-shaped embryo (**F**), the mutant embryo shows a globular-shaped embryo (**G**). Bars = 500 μm for (**B**) to (**E**) and 20 μm for (**F**,**G**).

To screen genes required for embryo development in *Arabidopsis thaliana*, we established a T-DNA insertion mutant library that contains one insertion site in the genomic DNA of *qrt1* plant background^16^,^17^. The T-DNA mutants contain the hygromycin resistance gene (Hyg^r^), and the LAT52::GFP gene. LAT52::GFP which is expressed in pollen, allows the identification of homozygous offspring from a given mutant plant^17^,^18^. The seed of *qrt1* is shown (Fig. 1B) for comparison. A heterozygous mutant with albino seed was isolated (Fig. 1C). The ratio of albino seeds was 16.35% (n = 981) out of the total seeds in the mutant siliques.

We identified the flanking sequence of the T-DNA insertion site of the mutated gene by thermal asymmetrical interlaced PCR^19^,^20^ and found that the T-DNA was inserted in the fourth exon of AT2g34480, a gene predicted to encode the ribosomal protein L18aB (RPL18aB), which is the member of the ribosomal L18ae/LX family protein^1^,^21^. The T-DNA insertion site is 113 bp upstream of the TAA end codon, and we designated the mutant *rpl18aB-1* (Fig. 1A). We also obtained an additional AT2g34480 allele (Flag-209B07), which we named *rpl18aB-2*, with a T-DNA insertion in the 5′ UTR, 23bp upstream of the ATG start codon (Fig. 1A). *rpl18aB-2/RPL18aB* plants showed a similar phenotype of aborted seeds as *rpl18aB-1/RPL18aB* during seed development (Fig. 1D). To further confirm that the phenotype of defective seed development was caused by loss of function of AT2g34480, a genomic *RPL18aB* gene was cloned in a kanamycin resistant vector and introduced into *rpl18aB-1/RPL18aB*. The seeds of transformed plants were screened on medium containing kanamycin and hygromycin, and seedlings showing both KAN^r^ and HYG^r^ were designated as complementation lines, transplanted to soil. Homozygous *rpl18aB-1* plants were obtained in the T2 progeny of genomic complementation lines (*rpl18aB-1/rpl18aB-1*, g-AT2g34480/g-AT2g34480) and normal seed development was restored (Fig. 1E). These results indicated that the AT2g34480 genomic fragments could successfully complement the *rpl18aB-1/*+ phenotype.

Normal ovules and albino ovules from the same silique in *rpl18aB-1/*+ were separated and observed by whole-mount clearing technique. In normal ovules, embryos had developed to the cotyledon stage (Fig. 1F), while in albino ovules the embryo remained in an abnormally globular-shaped stage (Fig. 1G). Both *rpl18aB-1/rpl18aB-1* and *rpl18aB-2/rpl18aB-2* mutant plants could not be recovered, suggesting the *rpl18aB-1/rpl18aB-1* and *rpl18aB-2/rpl18aB-2* embryos in albino ovules are embryo lethal. Unless specially mentioned, *rpl18aB* plant from here on means *rpl18aB/RPL18aB* heterozygous plant.

### *RPL18aB* is expressed in majority development process of Arabidopsis

**Figure 2:**
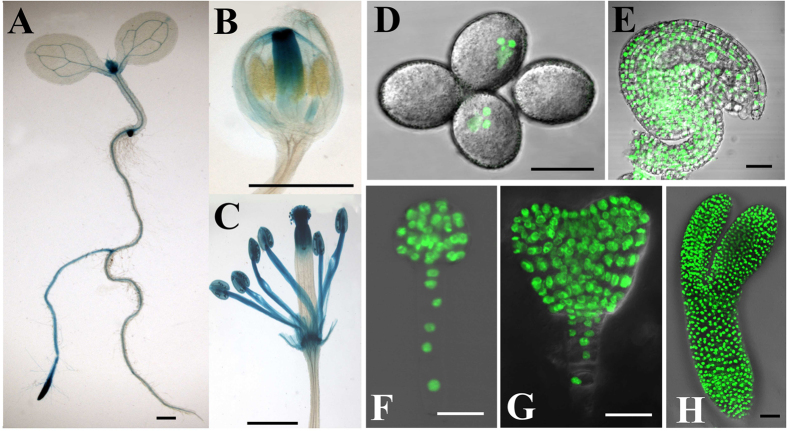
The expression pattern of *RPL18aB*. (**A**) p*RPL18aB::GUS* lines showed high GUS activity in the stem apex and root tip of seedlings, mainly in themeristem region of both parts. (**B**) Young flowers showed high GUS activity in pistil. (**C**) Mature flower showed GUS activity in stamens and upper pistil. (**D**) p*RPL18aB::H2B-GFP* lines showed GFP signal in nuclei of sperm cells and vegetative cell. (**E**) p*RPL18aB::H2B-GFP* lines showed GFP signal in nuclei of ovule cells, including female gametophytes. (**F**) p*RPL18aB::H2B-GFP* lines showed GFP signal in nuclei of globular-shaped embryocells. (**G**) p*RPL18aB::H2B-GFP* lines showed GFP signal in nuclei of early heart-shaped embryocells. (**H**) p*RPL18aB::H2B-GFP* lines showed GFP signal in nuclei of torpedo-shaped embryocells. Bars = 1 mm for (**A**) to (**C**) and 20 μm for (**D**) to (**H**).

To analyze the expression pattern of *RPL18aB*, we fused a 1.5 kb fragment of its native promoter to reporters GUS^22^ and H2B-GFP^22^ and produced p*RPL18aB::GUS* and p*RPL18aB::H2B-GFP*. The transgenic T2 progeny of p*RPL18aB::GUS* homozygous lines showed high GUS activity in the stem apex and the root tip of seedlings, mainly in the meristematic region (Fig. 2A). The vascular tissue of leaves also showed GUS activity. As for inflorescence developmental stages, GUS signal was observed in mature flowers but not in younger flowers (Fig. 2B,C). To clearly visualize the expression pattern of *RPL18aB* in reproductive organs, we studied the distribution of p*RPL18aB::H2B-GFP* and observed its expression in both male (Fig. 2D) and female gametophytes(Fig. 2E) and during embryo development (Fig. 2F–H).

### *rpl18aB* mutant reduces male/female gametophyte heredity

Our study showed that 16.35% aborted seeds were obtained from the siliques of *rpl18aB-1* plants, and 42.79% (n = 1138) of *rpl18aB-1* plants progeny seeds showed HYG^r^ resistance. The ratios are lower than the expected 25% and 66.7% ratios, respectively. These results suggest the existence of additional defects beyond the phenotype of abnormal seed development.

To clarify whether the additional defects are caused by the male or female part, we determined the mutant transmission efficiency through male/female gametophyte by reciprocal cross between *rpl18aB-1* plants and wild type plants. As shown in Table 1, the Hyg^r^/Hyg^s^ ratio of the crossed line’s seeds was 0.73:1 (Hyg^r^ accounted for 42.32% of the total) when *rpl18aB-1* plant was used as female parent, indicating a weak defect exists in the female gametophyte. However, the ratio dropped to 0.06:1 (Hyg^r^ accounted 5.74% of the total) when *rpl18aB-1* plant was used as male parent, demonstrating a stronger defect occurred in the male gametophyte.

### *rpl18aB* lowers pollen tube competitiveness

**Figure 3:**
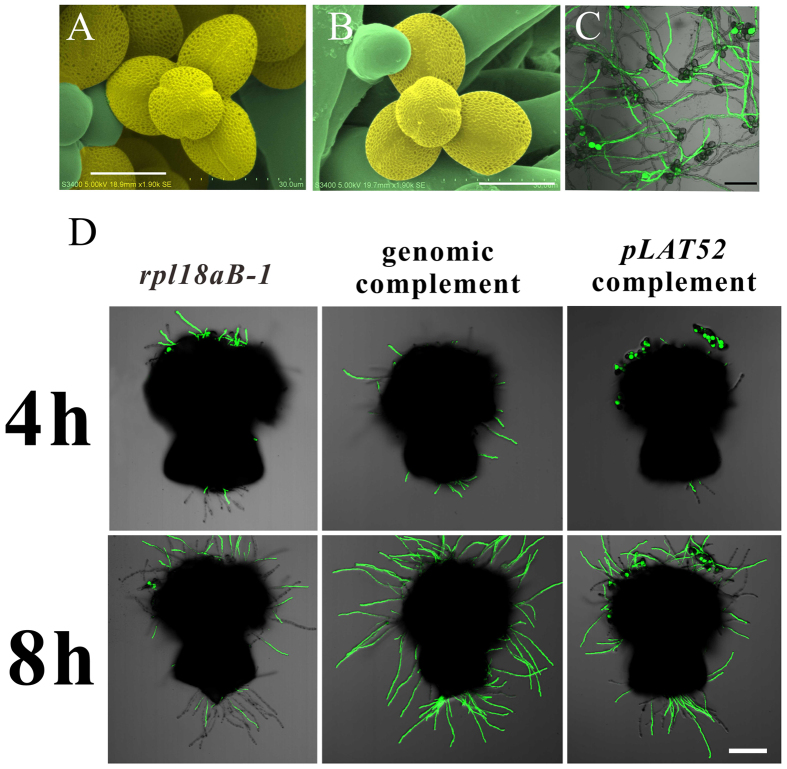
*rpl18aB-1* lowers pollen tube competitiveness. (**A**) Scanning electron micrographs showing pollen of wild type; the pollen was false-colored with yellow. (**B**) Scanning electron micrographs showing pollen of *rpl18aB-1*; the pollen was false-colored with yellow. (**C**) A CLSM image showing pollen tubes of both *rpl18aB-1* (marked by GFP) and wild type (without GFP) were germinated and grown to a similar length in *rpl18aB-1*. (**D**) CLSM images of pollen tubes growth for 4 h and 8 h on semi-*in vitro* conditions in *rpl18aB-1,* genomic complemented line and *pLAT52* complemented line respectively. *rpl18aB-1* pollen tubes were marked by GFP. Bars = 20 μm for (**A**) to (**B**) and 100 μm for (**C**) to (**D**).

We carried out assays to determine what defect occurred in the male gametophyte of *RPL18aB* mutant. Firstly, pollen grains of *rpl18aB-1* and wild-type plants were observed by scanning electron microscopy (SEM) to observe whether mutant pollen grains had structural defects, but no obvious morphological differences were observed (Fig. 3A,B). Secondly, pollen grains of *rpl18aB-1* were smeared on solid medium for pollen germination. After being cultured for 12 hours at 22 °C, pollen tubes of both mutant (marked by GFP) and wild type (without GFP) were germinated and grown to a similar length at the same time (Fig. 3C). Thirdly, we pollinated pollen grains of *rpl18aB-1* plants on wild type stigma. The pollinated stigma was cut from the style 1 hour later, and cultured on solid pollen germination medium. Pollen tubes could grow out of the cut end for several millimeters 4–8 hours after pollination, but few of them were *rpl18aB-1* pollen tubes (marked by GFP) (Fig. 3D). These results indicated that wild type pollen tube growth is much faster than mutant pollen tubes.

To test whether the decreased competitiveness of mutant pollen tubes could be recovered if specifically compensated for the defect of the pollen, we transformed *rpl18aB-1* plants with the *pLAT52::RPL18aB* construct. T1 *pLAT52* complementation lines (*rpl18aB-1/RPL18aB*, *pLAT52::RPL18aB*/+) were screened for further pollination experiments. The genomic complementation line served as a positive control. As expected, the genomic complementation line could recover the growth defect of mutant pollen tubes of *rpl18aB-1* (Fig. 3D). The ratio of GFP pollen tubes (*rpl18aB-1* background) that penetrated through style was higher in *pLAT52* complementation line compared with *rpl18aB-1* mutant (Fig. 3D).

It is possible that in *rpl18aB-1* plants few *rpl18aB-1* pollen tubes can enter the ovules because most ovules have been already occupied by wild type pollen tubes, leading to the very low observed transmission efficiency of Hyg^r^ through the male gametophyte. If this were true, decreasing the number of pollen grains used for pollination to ensure there are enough ovules to attract *rpl18aB-1* pollen tubes, the transmission efficiency of Hyg^r^ through the male gametophyte would be expected to increase. We tested this by the limited pollination technique and found that this ratio increased to 0.26:1 (Hyg^r^ accounted for 20.50% of the total) (Table 2, data of the first arrow). We further crossed wild type with the pollens of the four *pLAT52* complementation lines, respectively, to determine the transmission efficiency of Hyg^r^ through the male gametophyte. The results showed that the male transmission efficiency of Hyg^r^ increased from 5.74% (Table 1, data of the second arrow) to about 30% in all four *pLAT52* complementation lines (Table 2, data of the second to the fifth arrows). Together, these results confirmed that *rpl18aB* lowers pollen tube competitiveness in the style.

### Both radial and apical-basal patternings are disturbed in *rpl18aB-1/rpl18aB-1* embryo

**Figure 4:**
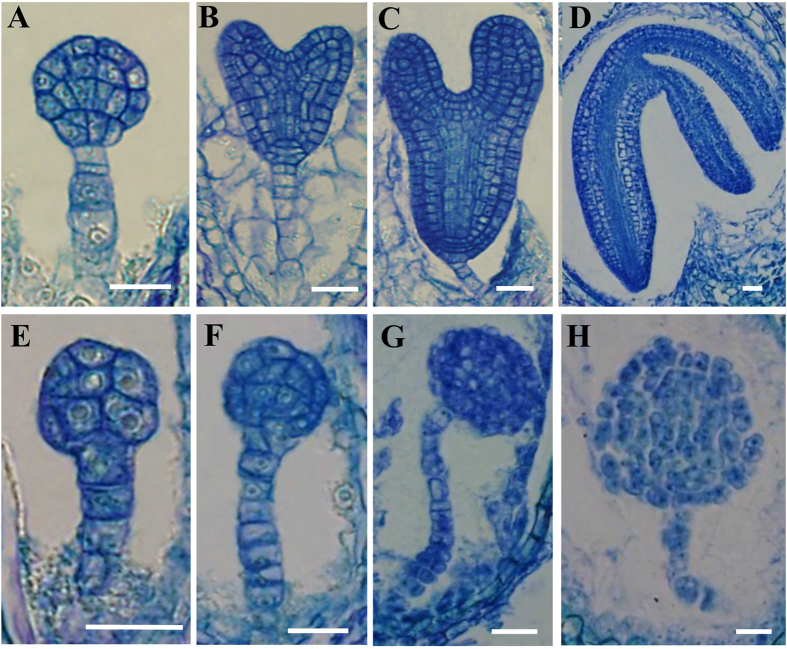
Pattern formation is perturbed in *rpl18aB-1/rpl18aB-1* embryos. (**A**–**D**) embryo development of wild type. In wild type ovules, embryogenesis developed from the globular stage (**A**), heart-shaped stage (**B**) torpedo-shaped stage (**C**), and finally to the matured cotyledon stage (**D**). (**E**–**H**) embryo development of *rpl18aB-1/rpl18aB-1* is delayed compared with wild-type embryos in the same slique of *rpl18aB-1*. Mutant embryos with undulated protoderm-like layer (**F**) or without recognizable protoderm cell layer (**G**). The *rpl18aB-1/rpl18aB-1* embryos were arrested at the globular stage and never underwent the transition stage for apical-basal pattern formation (**G**,**H**). Bars = 20 μm for (**A**) to (**H**).

To study embryogenesis in mutant ovules, a time series of sections of whole siliques were carried out with *rpl18aB-1* plants. In wild type ovules, embryogenesis developed from the globular stage, heart-shaped stage to torpedo-shaped stage, and finally to the matured cotyledon stage (Fig. 4A–D). During this process the radial pattern was first recognized with a clear protoderm layer surrounding the embryo (Fig. 4A). In the same silique, *rpl18aB-1/rpl18aB-1* embryos underwent a delayed pattern formation or failed to form this pattern (Fig. 4E–H). The cell division orientation was greatly altered, and thus the cell organization in *rpl18aB-1/rpl18aB-1* embryos was irregular, resulting in abnormal shapes of embryos with an undulated protoderm-like layer or without a recognizable protoderm cell layer (Fig. 4F,H). The *rpl18aB-1/rpl18aB-1* embryo development was arrested at the globular stage and never went through the transition stage for apical-basal pattern formation (Fig. 4F,H). Thus, in the mutant embryos both the radial and the apical-basal pattern formation are altered, and this leads to the abortion of embryogenesis and seed formation.

**Figure 5:**
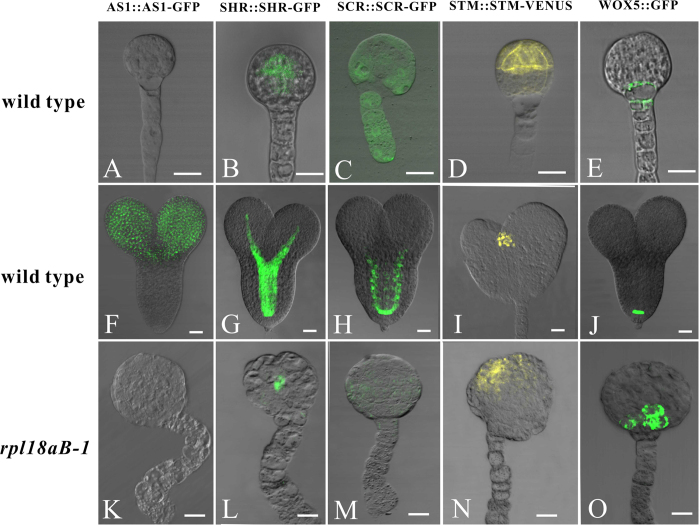
Cell-type specific expression pattern of marker genes were disrupted in *rpl18aB-1/rpl18aB-1* embryos. The upper arrow showed the globular embryos isolated from different marker lines. Both normal ovules and abnormal ovules in the same silique of different marker lines in *rpl18aB-1* could be found. The middle arrow showed isolated heart-stage embryos in normal ovules and the under arrow showed isolated *rpl18aB-1/rpl18aB-1* embryos in abnormal ovules.(**A**–**C**) AS1::AS1-GFP expression in wild-type globular embryo (**A**), heart-stage embryos (**B**) and *rpl18aB-1/rpl18aB-1* embryos (**C**). (**D**–**F**) SHR::SHR-GFP expression in wild-type globular embryo (**D**), heart-stage embryos (**E**) and *rpl18aB-1/rpl18aB-1* embryos (**F**). (**G**–**I**) SCR::SCR-GFP expression in wild-type globular embryo (**G**), heart-stage embryos (**H**) and *rpl18aB-1/rpl18aB-1* embryos (**I**). (**J**–**L**) STM::STM-VENUS expression in wild-type globular embryo (**J**), heart-stage embryos (**K**) and *rpl18aB-1/rpl18aB-1* embryos (**L**). (**M**–**O**) WOX5::GFP expression in wild-type globular embryo (**M**), heart-stage embryos (**N**) and *rpl18aB-1/rpl18aB-1* embryos (**O**). Bars = 20 μm for (**A**) to (**O**).

Although morphologically the *rpl18aB-1/rpl18aB-1* embryos were blocked at the globular stage, the embryos actually continued to undergo different rounds of cell division, resulting in big globular-shaped embryos of approximately one hundred cells (Fig. 4H). To further detect whether the pattern formation is indeed disturbed in *rpl18aB-1/rpl18aB-1*, we constructed embryo markers and transformed them into *rpl18aB-1* plants. AS1::AS1-GFP, which is expressed mainly in the cotyledon primordia and growing point^23^ (Fig. 5B), SHR::SHR-GFP^24^ and SCR::SCR-GFP^25^, which are expressed in the central area of embryo proper (Fig. 5D,E,G,H), In addition, a shoot apical meristem marker line, STM::STM-VENUS^26^ (Fig. 5J,K) and the root tip marker line WOX5::GFP line^27^ (Fig. 5M,N) were crossed with *rpl18aB-1* respectively. All positive transgenic lines and hybrids were screened. When wild type embryos developed to the heart-shaped embryo stage, both normal and mutant embryos in the same silique were isolated to observe the expression pattern of the tissue specific markers. In addition, wild type globular embryos in these marker lines were isolated. In wild type, AS1::AS1-GFP was not expressed in the globular embryo but expressed in heart-shaped embryos, while there was no AS1::AS1-GFP expression in *rpl18aB-1/rpl18aB-1* embryos (Fig. 5A–C). These results further indicated that the *rpl18aB-1/rpl18aB-1* embryos were arrested at the globular stage and never underwent the transition stage for apical-basal pattern formation. As shown in Fig. 5, the other cell-type specific expression pattern of marker genes were disrupted in *rpl18aB-1/rpl18aB-1* embryos (Fig. 5F,I,L,O). These observations further suggested that pattern formation in the mutant embryo was disturbed.

### Auxin gradient is altered in *rpl18aB-1/rpl18aB-1* embryo

**Figure 6:**
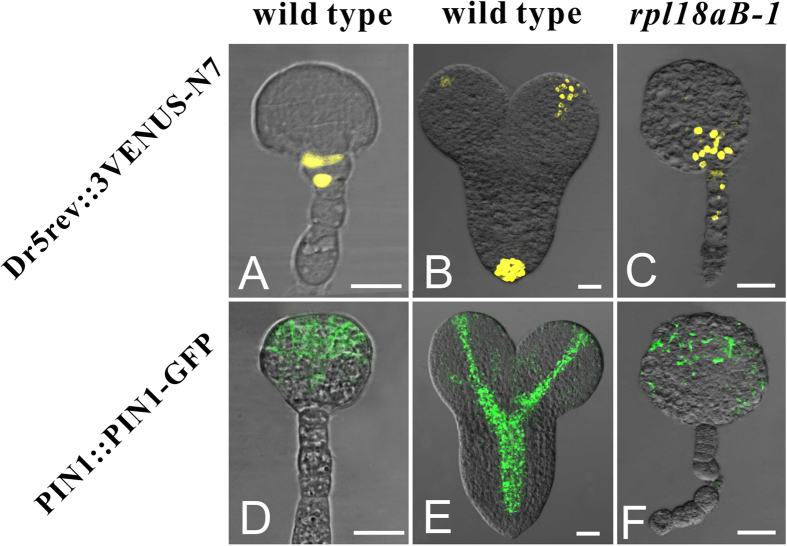
Auxin gradient was altered in *rpl18aB-1/rpl18aB-1* embryos. (**A**–**C**) DR5rev::3VENUS-N7 expression in wild-type globular embryo (**A**), heart-stage embryos (**B**) and *rpl18aB-1/rpl18aB-1* embryos (**C**). (**D**–**F**) PIN1::PIN1-GFP expression in wild-type globular embryo (**D**), heart-stage embryos (**E**) and *rpl18aB-1/rpl18aB-1* embryos (**F**). Bars = 20 μm for (**A**) to (**F**).

It is well established that auxin is important for pattern formation of embryo^28^,^29^,^30^,^31^,^32^,^33^. As *rpl18aB-1* embryo could not develop into heart stage, we reasoned that the auxin distribution might be impaired in these mutants. Firstly a cellular auxin levels marker line DR5rev::3VENUS-N7^34^, was crossed with *rpl18aB-1*. Fluorescence was observed at the base of embryo proper during the globular stage and mainly at the tips of cotyledons and in the quiescent center during the heart stage in wild-type (Fig. 6A,B). However, in globular-shaped *rpl18aB-1/rpl18aB-1* embryos, the distribution of DR5rev::3VENUS-N7 was extended from the embryo proper bottom to uppermost suspensor. These results indicate that the auxin gradient is altered in *rpl18aB-1/rpl18aB-1* embryos (Fig. 6C).

The Arabidopsis auxin polar transport carrier PIN-FORMED 1 (PIN1) is responsible for the establishment of an auxin gradient and expressed in the apical part of embryo^27^,^29^,^31^,^35^. To visualize the localization of PIN1 in the embryos, we used PIN1::PIN1-GFP plants^27^ and crossed them with *rpl18aB-1* plants. In wild type embryos, PIN1 mainly localized in the embryo proper at the globular stage, and in vasculature and hypocotyls of developing cotyledons at the heart stage (Fig. 6D,E). In *rpl18aB-1/rpl18aB-1* late globular embryos, PIN1 presented a disorganized distribution (Fig. 6F). These findings indicated the auxin polar transportation pathway is affected in *rpl18aB-1/rpl18aB-1* embryos, leading to a disordered auxin distribution and abnormal pattern formation.

### Specific expression of RPL18aB in different tissues of mutant embryo proper could not rescue the phenotype

**Figure 7:**
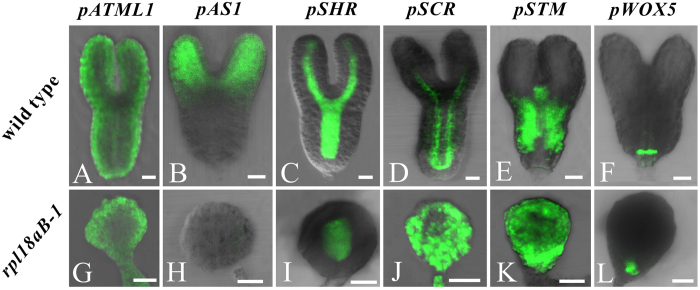
Specific expression of RPL18aB in different tissues of mutant embryo proper could not rescue the phenotype. (**A**,**G**) pATML1::RPL18aB-EGFP expression in wild-type torpedo-shaped embryo (**A**) and *rpl18aB-1/rpl18aB-1* embryos (**G**). (**B**,**H**) pAS1::RPL18aB-EGFP expression in wild-type torpedo-shaped embryo (**B**) and *rpl18aB-1/rpl18aB-1* embryos (**H**). (**C**,**I**) pSHR::RPL18aB-EGFP expression in wild-type torpedo-shaped embryo (**C**) and *rpl18aB-1/rpl18aB-1* embryos (**I**). (**D**,**J**) pSCR::RPL18aB-EGFP expression in wild-type torpedo-shaped embryo (**D**)and *rpl18aB-1/rpl18aB-1* embryos (**J**). (**E**,**K**) pSTM::RPL18aB-EGFP expression in wild-type torpedo-shaped embryo (**E**) and *rpl18aB-1/rpl18aB-1* embryos (**K**). (**F**,**L**) pWOX5::RPL18aB-EGFP expression in wild-type torpedo-shaped embryo (**F**) and *rpl18aB-1/rpl18aB-1* embryos (**L**). Bars = 20μm for (**A**) to (**L**).

As described above, specific tissue layers of mutant embryos were abnormal. We reasoned that the abortion of *rpl18aB* homozygous embryos may be the result of the suppression of a certain tissue layer responsible for the formation of heart-shape embryo. If it were true, specifical rescue of the relevant cell layers would recover the defect in pattern formation. To test this hypothesis, we constructed seven expression vectors and transformed them to *rpl18aB-1* plants independently. These constructs include p*ATML1::RPL18aB-EGFP*, which use the promoter of epidermal cell layer specific gene *ARABIDOPSIS THALIANA MERISTEM LAYER1* (*ATML1*)^36^,^37^ to drives the expression of RPL18aB in the epidermal cell layer of the embryo (Fig. 7A); p*AS1::RPL18aB-EGFP*, which drives the expression of RPL18aB in the growing ends of the cotyledon (Fig. 7B); p*SHR::RPL18aB-EGFP* and p*SCR::RPL18aB-EGFP*, which drive the expression of RPL18aB in the central area of embryo proper (Fig. 7C,D); p*STM::RPL18aB-EGFP*, which drives the expression of RPL18aB in the shoot apical meristem (Fig. 7E); p*WOX5::RPL18aB-EGFP*, which drives the expression RPL18aB in the hypophysis (Fig. 7F). All the six constructs were unable to recover the aberrant embryogenesis mutant phenotype (Fig. 7G–L). Among them, pATML1::RPL18aB-EGFP, pSHR::RPL18aB-EGFP, pSCR::RPL18aB-EGFP, pSTM::RPL18aB-EGFP and pWOX5::RPL18aB-EGFP were detected in the *rpl18aB-1/rpl18aB-1* embryo proper but with ectopic expression (Fig. 7G,I–L). For the construct pAS1::RPL18aB-EGFP, it was not expressed in the predicted embryo proper domain in *rpl18aB-1/rpl18aB-1* embryo (Fig. 7H). These results indicate that recovery of the specific tissue or cell layer are not sufficient to support normal pattern formation.

## Discussion

Embryogenesis is the process of pattern formation. In Arabidopsis, the zygote divides asymmetrically following fertilization, produces an apical cell and a basal cell, and then further develops into the embryo proper and suspensor domains^38^,^39^. In the embryo proper domain, a radial pattern could be observed as early as at the 16-celled embryo stage, which is indicated by a clear protoderm and the enclosed provescular and ground tissue^39^,^40^. When the embryo develops into the globular stage, the primordia of cotyledons are generated, which morphologically marks the establishment of apical-basal pattern^41^.

The differentiation of protoderm cells involves some tissue specific expressed genes. *ATML1*is specifically expressed in the apical cell lineage, and after the 16-cell embryo stage it only expresses in protoderm cells^37^. *PROTODERMAL FACTOR2* (*PDF2*) has a similar expression pattern. Both genes encode START domain containing transcription factors and positively regulate epidermal cell differentiation^42^,^43^. Although other genes are also involved in radial pattern formation of the embryo, such as *FASS, SCR* and *WOL*^24^,^25^,^44^, the mechanisms determining the cell fate of the protoderm and other radial layers of the embryo remain to be elucidated.

Apical-basal pattern is gradually established after the globular embryo stage. WUS is detected first in the 16-cell embryo, and is critical for apical meristem formation^45^. PINOID and ENHANCER OF PINOID are required for the auxin accumulation in apical cells and cotyledon formation^46^,^47^,^48^,^49^. CUP SHAPED COTYLEDON is critical to suppress the apical meristem between cotyledon primordia and thus to separate them^50^,^51^,^52^,^53^. These studies revealed the existence of a very complex regulatory mechanism for embryo pattern formation, and highlight the remaining need for future investigations to unravel this regulatory network.

In our study, we found that *rpl18aB* embryos undergo developmental arrest at the globular stage, with possible, secondary pattern formation defects. In fact, as early as at the 8-celled embryo stage irregular cell division orientations could be observed, resulting in disorganization of embryo cells and the differentiation of different domains in both radial and apical-basal structures of embryos, suggesting that an early step is interrupted and this early developmental process is critical for both the radial and apical-basal pattern formation. Interestingly, based on previous works, we carried out cell type-specific complementation of RPL18aB in mutant embryos. We used p*ATML1::RPL18aB-EGFP* for epidermal cell layer, p*AS1::RPL18aB-EGFP* for cotyledon primordia and growing end, p*STM::RPL18aB-EGFP* for shoot apical meristem, p*WOX5::RPL18aB-EGFP* for hypophysis, p*SHR::RPL18aB-EGFP* and p*SCR::RPL18aB-EGFP* for the central area of the embryo proper specific complementation respectively. These complementations were expected to recover different cell types and thus offer the opportunity to test the role of each of these cell types in embryo pattern formation. However, the results showed that none of these complementations could recover the failed embryo pattern formation phenotype. These results indicated that RPL18aB might play an essential role in the cell fate determination during early embryogenesis. Since most of these marker genes are not expressed at the very early stage, our results suggest that the first several divisions of apical cell lineage are critical for the subsequent embryo pattern formation. Even though some of the cell types were recovered by the cell type specific supplementation and the later embryo development could be improved in some way, the pattern formation phenotype could not be fully recovered since early cell division was irregular. How these early cell divisions could change cell fate is an interesting question and should be elucidated in future studies.

Recently, ribosomal proteins were found to play a multifunctional role in plant development^3^,^4^,^5^,^6^,^7^,^8^,^9^, and their role in sexual plant reproduction has drawn a great attention^11^,^12^,^13^,^14^. During embryogenesis, mutations in several cytoplasmic ribosomal proteins, such as S6, S11, L2, L8, L23, L19 and L40, results in embryo lethality, indicating their critical role in embryogenesis. These ribosomal proteins mainly function in the later stages of embryogenesis^15^. In the present study, we report a new ribosomal protein RPL18aB that also functions in the early stage of development and specifically involved in early cell division orientations. The mutant *rpl18aB* offers an opportunity to test the influence of early cell division on the cell fate determination and subsequent embryo pattern formation. Future studies should focus on elucidating the relationship between this ribosomal protein, the control of cell division orientation and to the cell fate decision.

## Materials and Methods

### Plant materials and growth conditions

The *rpl18aB-1* allele was isolated from our mutant library with Hyg^r^ (Wu *et al*.^17^). The *rpl18aB-2* (Flag-209B07) was obtained from INRA (Versailles, France), STM::STM-VENUS (CS-67930) and DR5rev::3VENUS-N7(CS-67931) were obtained from ABRC, Ohio, USA. Marker lines WOX5::GFP and PIN1::PIN1-GFP were provided by Dr. Jian Xu (Temasek Life Sciences Laboratory, Singapore). Seeds were surface sterilized with 20% bleach for 10 min, and washed three times with sterile distilled water. Seeds were stratified for 3 days at 4 °C, and then sown on 1/2 MS plates with 1.0% (w/v) sucrose. Agar plates were placed in a growth room with a photoperiod of 16 h light/8h dark. For kanamycin selection, 50 mg/L of kanamycin (Sigma) was supplemented to the media. Similarly, 50 mg/L of hygromycin (Roche) was added for hygromycin selection. Plants were grown in soil in a greenhouse under long-day conditions (16 h light/8 h dark) at 22 °C.

### Cloning of the T-DNA flanking sequence of the *rpl18aB-1*

The T-DNA flanking sequence in the *rpl18aB-1* mutant was cloned by TAIL-PCR (Liu *et al*.^19^). The authenticity of the cloned sequence was confirmed by PCR using two pairs primers located around the T-DNA border (*rpl18aB-1-T1:* GCCCCTCTCTTGACTAATGTAATC; LB-S: CCAAAATCCAGTACTAAAATCCAG) and (*rpl18aB-1-T2*: CATGAACAAGTTGGGCTTGTTGG; LB-S: CCAAAATCCAGTACTAAAATCCAG) respectively.

### Vector construction and plant transformation

Plasmid P092 and P094 were produced as previous described^17^. To generate the genomic complementation construct, A 3250-bp wild-type genomic sequence containing the AT2g34480 gene, 1690-bp upstream of the ATG codon and 314-bp downstream of the TAG codon sequences, was PCR-amplified with primers *RPL18aB-F1:* NNNNTCTAGATTGGTGATCAAAGTCAATACTCATG and *RPL18aB-R1:* NNNNGCGGCCGCGTTTGAAGGCAAAATTACAGTAGAG from genomic DNA and then was cloned into the P092 plasmid. To investigate the expression pattern of *RPL18aB*, we first cloned the *GUS* fragment with the *NOS* terminator from p*CAMBIA1305* and then introduced them into the plasmid PART27 to generate P093. We then cloned the H2B coding fragment (AT5G22880) and cloned it into P094to generate P095. The *RPL18aB promoter* was amplified with primers *RPL18aB-F1 and RPL18aB-F1-R2:* NNNNAAGCTTTTCGTCTGGAGAGAGACAATG and put upstream of GUS in P093 and H2B-GFP in P095 to generate p*RPL18aB*::*GUS* and p*RPL18aB*::*H2B-GFP*. To produce the embryo markers of AS1::AS1:GFP, SHR::SHR:GFP and SCR::SCR:GFP constructs, the promoter and CDs sequences of AS1, SHR, SCR were amplified with primers shown in Table 3 and cloned into p094 respectively. To specifically express *RPL18aB* in the pollens and embryos respectively, the *RPL18aB* ORF was amplified with primers *RPL18aB-CDS1:* NNNNGGTACCGCCACCATGGGTGCTTTCAGGTTTCACC and *RPL18aB-CDS2:* NNNNAAGCTTCATGAACAAGTTGGGCTTGTTG from the cDNA of flower and cloned into p094 to generate a *RPL18aB-EGFP* construct. Then the promoter of AS1, ATML1, SHR, SCR, WOX5, STEM and LAT52 were amplified with primers shown in Table 3 and fused upstream of *RPL18aB-EGFP* respectively.

All constructs were transformed into *Agrobacterium tumefaciens* strain GV3101, and then transformed into Arabidopsis plants by the floral dip method^54^.

### Phenotype Characterization of embryo development

To prepare cleared whole-mount seeds, seeds were dissected from siliques and cleared using chloral hydrate solution^55^. The cleared seeds were placed under a microscope (Olympus) fitted with differential interference contrast optics for imaging.

Fertilized siliques at different developmental stages were fixed overnight in 4% (v/v) formaldehyde, dehydrated in a graded ethanol series (10%, 30%, 50%, 70%, 80%, 90%, 95% and 100% twice), transferred to xylene and embedded in Sigma’s paraplast. Semi-thin sections (10 μm) were cut on a rotary microtome (American optical, USA), and heat fixed to glass slides. Sections were stained with toluidine blue before removal of paraffin with xylene (20 min twice). Bright-field images of the ovule cross-sections were taken using an Olympus microscope.

### Pollen germination assays

*In vitro* pollen germination was performed according to Ye *et al*.^56^. Pollen was harvested from newly opened flowers and placed onto the pollen germination medium consisting 1 mM CaCl_2_, 1 mM Ca(NO_3_)_2_, 1 mM MgSO_4_, 0.01% (w/v) H_3_BO_3_, and 18% (w/v) sucrose solidified with 0.8% (w/v) agar, pH 7.0. The plates were cultured at 28 °C at high humidity. Semi-*in vitro* pollen growth assay was performed as previously described^57^. After hand-pollination, pistils were cut at the shoulder region of the ovaries. Cut pistils were incubated on pollen tube growth medium at 28 °C.

### Scanning Electron Microscopy

For scanning electron microscopy examination, the opened flowers of the *qrt1* and *rpl18aB-1* were dissected and the dehiscent anthers were coated with 8-nm gold. Observations and images were performed with a S-3400N scanning electron microscope (HITACHI).

### Histochemical analysis of GUS activity

The histochemical analysis of GUS activity was performed according to Vielle-Calzada *et al*.^58^. Plant tissues were incubated at 37 °C in GUS staining solution [2 mM 5-bromo-4-chloro-3-indolyl glucuronide (X-Gluc) in 50 mM sodium phosphate buffer, pH 7.0] containing 0.1% Triton X-100, 2 mM K_4_Fe(CN)_6_ and 2 mM K_3_Fe(CN)_6_. The stained tissues were then transferred to 70% (v/v) ethanol solution. Samples were mounted with traditional clearing solution and placed under a microscope (Olympus) fitted with differential interference contrast optics for imaging.

### Embryo separation and CLSM microscopy

For fluorescent marker line analysis, ovules of Arabidopsis at specific development periods were collected and put on a 30 mm diameter culture plate with a drop of 10% glycerin added with 80 mM sorbitol. A sharp capillary glass tube was used as the dissection tool to extract the embryos from maternal tissues. The isolated embryos were visualized using a FV1000 confocal laser-scanning microscope (CLSM; Olympus). GFP fluorescence was detected with excitation at 488 nm.

## Additional Information

**How to cite this article**: Yan, H. *et al*. Ribosomal protein L18aB is required for both male gametophyte function and embryo development in *Arabidopsis*. *Sci. Rep.* **6**, 31195; doi: 10.1038/srep31195 (2016).

## Figures and Tables

**Table 1:** Segregation of the *rpl18aB*-1 Mutation in Selfed and Reciprocally Backcrossed Offspring Populations.

Parental Genotype		Progeny Genotype		
Male	Female	Hyg^r^	Hyg^s^	Hyg^r^ Ratio
wild type	*rpl18aB-1*/RPL18aB	482	657	42.32%
*rpl18aB-1*/RPL18aB	wild type	38	624	5.74%
*rpl18aB-1*/RPL18aB	*rpl18aB-1*/RPL18aB	1270	1856	40.63%

**Table 2:** Segregation of the *rpl18aB-*1 Mutation in Backcrossed Offspring Populations under Limited Pollination Conditions and *pLAT52* complementation lines.

Parental Genotype		Progeny Genotype		
Male	Female	Hyg^r^	Hyg^s^	Hyg^r^ Ratio
*rpl18aB-1*/RPL18aB, L	WT	230	892	20.50%
LAT52::RPL18aB-1	WT	174	411	29.74%
LAT52::RPL18aB-2	WT	159	412	27.85%
LAT52::RPL18aB-3	WT	178	425	29.72%
LAT52::RPL18aB-4	WT	262	533	32.96%

L refers to limited pollination. LAT52::RPL18aB series are *pLAT52* complementation lines.

**Table 3:** Primers.

Name	Primers (5′ to 3′)
SCR CDS-1	NNNNCCTAGGATGGCGGAATCCGGCG
SCR CDS-2	NNNNGAATTCAGAACGAGGCGTCCAAGCT
SHR CDS-1	NNNNGCGGCCGCATGGATACTCTCTTTAGACTAGTCAGTCTCC
SHR CDS-2	NNNNGAATTCACTAGCCCAAACCACCGGCT
AS1 CDS-1	NNNNGCGGCCGCATGAAAGAGAGACAACGTTGGAGTG
AS1 CDS-2	NNNNGAATTCGGGGCGGTCTAATCTGCAAC
LAT52 promoter-1	NNNNGAGCTCTGTCGACATACTCGACTCAGAAG
LAT52 promoter-2	NNNNGGTACCTTTAAATTGGAATTTTTTTTTTTGG
SCR promoter-1	NNNNGCGGCCGCGATTTTGCTGGTGTTGAATGGAT
SCR promoter-2	NNNNCCTAGGGGAGATTGAAGGGTTGTTGGTC
ATML1 promoter-1	NNNNCCAACGCGTTGGGTGTTTACATTGATTAGTAGGCTGC
ATML1 promoter-2	NNNNGCGGCCGCGATGATGATGGATGCCTATCAAT
SHR promoter-1	NNNNCCAACGCGTTGGATGGGGAAGATGTTTTCTCCAAGTA
SHR promoter-2	NNNNGCGGCCGCTAATGAATAAGAAAATGAATAGAAG
AS1 promoter-1	NNNNCCAACGCGTTGGGAGAAATATATTAGGCAATCAAACGG
AS1 promoter-2	NNNNGCGGCCGCCTCCTACTCCTCCTGACATCACTTC
WOX5 promoter-1	NNNNCCAACGCGTTGGCTTGCAAAATTTGATCGTCGTAC
WOX5 promoter-2	NNNNGCGGCCGCGTTCAGATGTAAAGTCCTCAACTGTT
STM promoter-1	NNNNGCGGCCGCACGTTATCCTAATTTTGTTTATCCTA
STM promoter-2	NNNNCCTAGGCTTCTCTTTCTCTCACTAGTATTATTATTCA
